# Accelerating maturation of Chinese rice wine by using a 20 L scale multi-sweeping-frequency mode ultrasonic reactor and its mechanism exploration

**DOI:** 10.1016/j.ultsonch.2025.107229

**Published:** 2025-01-12

**Authors:** Zhuofan He, Furong Hou, Yansheng Du, Chunhua Dai, Ronghai He, Haile Ma

**Affiliations:** a School of Electrical and Information Engineering, Jiangsu University, 301 Xuefu Road, Zhenjiang, Jiangsu 212013, China; b School of Food and Biological Engineering, Jiangsu University, 301 Xuefu Road, Zhenjiang, Jiangsu 212013, China; c Institute of Food and Nutrition Science and Technology, Shandong Academy of Agricultural Sciences, 202 Gongye North Road, Jinan, Shandong 250131, China; d School of Environmental and Safety Engineering, Jiangsu University, 301 Xuefu Road, Zhenjiang, Jiangsu 212013, China

**Keywords:** Chinese rice wine, Ultrasonic reactor, Storage, Acoustic cavitation, Maturation

## Abstract

The formation of flavor in traditional Chinese rice wine requires a long aging process. To accelerate the maturation of rice wine, a 20 L scale multi-sweeping- frequency mode ultrasonic reactor was employed in this study to explore the promoting effects. Rice wines were subjected under 10 combined types of sonication treatments with 20/28/40 kHz in single/double/triple frequencies, and in fixed or sweeping modes, respectively. Then samples were aged in room temperature for up to 180 days. A 7.3 % increase of total esters content was observed in rice wine sample after treated by a fixed 40 kHz ultrasonication with 50 W/L intensity at 30 °C for 15 min, compared with the untreated sample. After sonication and stored for six months, 286.78 % increase of the volatile esters was found, compared to rice wine without ultrasoinc treatment and stored at same condition for same time. And the total volatile alcohol substances and total volatile aldehydes in rice wine decreased by 12.95 % and 67.46 %, while the total volatile acids increased by 17.11 %, respectively. The research results also demonstrated that ultrasonic induced free radicals accounted for the variations of rice wine properties. And the correlation between the acoustic cavitation and the flavor formation was also observed.

## Highlights

• Employ a 20 L multi-sweeping-frequency ultrasonics reactor to accelerate maturation of rice wine.
• A fixed 40 kHz ultrasonication on 50 W/L intensity at 30 oC for 15 min sonication is optimal.
• Sonication increase 286.78% volatile esters compared to control after stored for six months.
• Ultrasonic induced free radicals account for the promotion of rice wine properties.

## 1 Introduction

Chinese rice wine, grape wine and beer, are the world's three ancient wines [1]. Taken rice, wheat, corn, millet as the main raw materials, Chinese rice wine was made by cooking, saccharification and fermentation, pressing, filtering, sterilization, storage and blending. However, the newly brewed rice wine generally tastes astringency, flavourless and bitterness, and the ingredients in it are unstable. Therefore a long time of natural maturation (so called “aging”) process is necessary to promote the oxidation, esterification, condensation. The traditional aging method for wines was also called natural aging: storing fermented fresh wine in oak barrels or other container for storage for several months to several years, and a series of physical and chemical reactions among molecules of the wine, thereby improving the quality and flavor [2], [3]. It was found that the main volatile flavor components included seven esters (ethyl acetate, ethyl lactate, ethyl caproate, ethyl phenylacetate, phenylethyl acetate, ethyl benzoate and diethyl succinate), three higher alcohols (including isobutanol, isoamyl alcohol and phenylethanol), acetaldehyde, furfural and naphthalene [4], [5]. Whereas the natural maturation process is time-consuming, with high costs, and the amount of flavor compounds are still not meet the requirement. Therefore, it is urgently needed of some innovative accelerating technologies for maturation in traditional Chinese rice wine production. Till now, different accelerating technologies for the aging of wine were explored, which include micro-oxygenation, the physical and chemical methods, including ultrahigh pressure, pulsed electric field, ultrasound field, micro-wave field, etc., and coupling technologies among the above ones [5], [6].

In recent years, non-thermal technologies, especial ultrasound technology has been recognized as a prospective technology in shortening the wine aging process by accelerating some types of chemical reaction rates significantly [7], [8], [9], [10], [11], [12]. Saterlay et al. [13] found that ultrasonic wave could create an acoustic cavitation of microbubbles. Acoustic cavitation (formation, growth and implosive collapse of bubbles) leads to energy accumulations in hot spots, and high temperature and high pressure can be generated, which produces very high shear energy waves and turbulence and generates free radicals (in the cavity), leading chemical polymers to be broken into numerous mist particles (subparticles) and then recombine as new polymers with good flavor body [14], [15], [16], [17].

Researchers have noticed this novel physical processing technology’s application in wine production. Although sonication was not able to significantly influence the titratable acidity of various kinds of wines (such as maize, rice and white grape wine) [18], [19], [20], ultrasonic treatment can promote sensory characteristics and accelerate the formation of the flavor substances, such as esters and olefins [20], [21], [22]. Wines sonicated for 20 min had more complex sensory characteristics than the untreated one [20], this probably dues to the release of some components responsible for wine aroma and flavour from cells by cavitation. Soria et al. [21] reported that the ultrasonic wave in a frequency range of 16–60 kHz was able to accelerate oxidation, polymerization and condensation of alcohol, aldehydes, esters and olefins in wines. Zhang et al. [22] found that ultrasound could trigger the generation of 1-hydroxylethyl free radicals into red wine, which might be related to a chain of chemical reactions in wine.

The maturation of rice wine is a long time process, whereas the sonication’s benefit for the maturation of rice wine during storage are rarely reported. The influence of ultrasonic induced free radical chemical reactions can last for a long time. Therefore, it is worth studying the accelerating effect and its mechanism of ultrasound on maturation of rice wine during storage.

Until now, researches on studying the effect of ultrasound to wine maturating have achieved some valuable progress, but they mainly involved in laboratory ultrasonic equipment, there are very few reports on scaling up study.

Based on former studies of ultrasonic treatments on wines, the objective of this study was to employ a 20 L scale multi-sweeping-frequency mode ultrasonic reactor for accelerating the maturating of Chinese rice wine, to explore the variations of flavor and volatile substances during different times from 0 to 180 days’ storage, and to clarify the effects of ultrasonic induced free radicals and cavitation on maturation process of rice wine.

## 2 Materials and methods

### 2.1 Materials

Newly brewed semi-dry Chinese rice wine was provided by Hengshun Wine Co., Ltd. (Zhenjiang, China), containing 6.35 g/L, 13.61 g/L, 3.41 g/L and 0.81 g/L of total sugars, total acids, total esters and amino acids nitrogen, respectively. Oxalic acid, tartaric acid, malic acid, ketoglutaric acid, lactic acid, citric acid, acetic acid, succinic acid and acetonitrile were chromatographic pure, Sinopharm Chemical Reagent Co., Ltd. (Shanghai, China). Styrene was purchased from Sigma. All solvents and chemicals (Sinopharm Co., Ltd.) used in this study were of analytical grade.

### 2.2 Ultrasound treatment

**Fig. 1:**
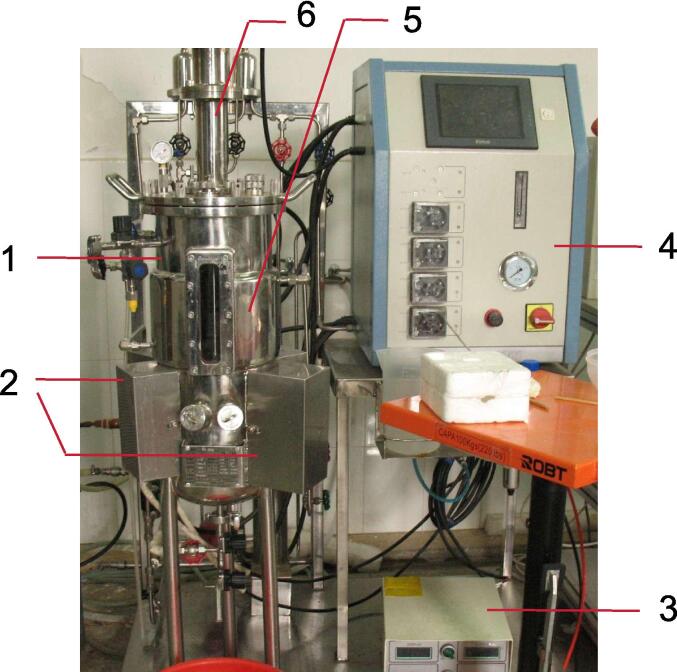
The 20 L Multi-Sweeping-Frequency Mode Ultrasonics Reactor 1- Reactor tank; 2- Sonicators chambers; 3- Ultrasonic generator; 4- Reactor Control Center; 5- Jacketed water bath temperature control system; 6- Mixing device.

A 20 L Multi-Sweeping-Frequency Mode Ultrasonics Reactor (Fig. 1, made by Zhenjiang Five Pines Biological Technology Co. Ltd, Zhenjiang, China) was used for sonication treatment. The reactor has a stainless steel tank with a jacketed water bath and temperature control system. The tank is coupled with three sonicator chambers (in each chamber, 6 ultrasonic transducers were adhered onto the outer wall surface of the tank). And ultrasonic generators connected with the transducers. The ultrasonic generator can generate 20, 28 and 40 kHz frequencies ultrasonic signal for each corresponding transducer on the tank’s outer wall. Each frequency ultrasound can work in a ± 2 kHz sweeping frequency mode, i.e., 20 ± 2, 28 ± 2 and 40 ± 2 kHz, respectively.

For each treatment, 15 L of Chinese rice wine was pumped into the reactor tank and heated to 30 °C, and then the ultrasonic generator was started for sonication treatment.

Based on former reports [12], [21], [22], [23], [24], [25], ten different frequency combinations of sonication treatments were carried out in this study, i.e., (1) single-sweeping frequency mode: 20 ± 2 kHz, 28 ± 2 kHz, 40 ± 2 kHz; (2) single-fixed frequency mode: 20 kHz, 28 kHz, 40 kHz; (3) double-fixed combined frequencies mode: 20/28 kHz, 20/40 kHz, 28/40 kHz; and (4) triple-fixed combined frequencies mode: 20/28/40 kHz. The conditions were based on the research of some studies [12], [21], [22], [23], [24], [25]. For each treatment, total ultrasonic power density was 50 W/L (i.e., output power of single frequency treatment is 750 W for 15 L wine, each frequency’s output power of dual frequency treatment is 375 W, and 250 W for each frequency’s output power in triple frequency treatment), and sonicating for 15 min at 30 °C. All sonication treatments were carried out in triplicate. After taken samples for analysis, the treated wines were stored for different days under room temperature for 7, 30, 90 and 180 days in polyethylene pots (for 7 days storage, average high temperature 19 °C and average low temperature 11.1 °C; for 30 days storage, average high temperature 23 °C and average low temperature 13 °C; for 90 days storage, average high temperature 25.66 °C and average low temperature 16.66 °C; for 180 days storage, average high temperature 29.55 °C and average low temperature 21.92 °C).

### 2.3 Total acids and total esters contents analysis

The acids in the rice wine have the effect of buffering with the other ingredients, and they can also coordinate other flavor and taste components. Therefore, there is the saying of “No acid and no flavor”. The esters of Chinese rice wine are mainly ethyl acetate and ethyl lactate. Esters have an important impact on the aroma and taste of rice wine, which can enrich the flavor and fragrance of the wine. The amount of total esters content directly reflects the degree of esterification in the process of wine aging, is an important indicator of the aging process.

The total acids and total esters contents were tested according to Chinese National Standard GB/T 13662-2008 and GB/T 10345.5-1989, respectively.

### 2.4 The determination of organic acids

The contents of eight organic acids (oxalic acid, tartaric acid, malic acid, citric acid, ketoglutaric acid, succinic acid, acetic acid, lactic acid) of rice wine were analyzed by High Performance Liquid Chromatography (HPLC, LC-20AT, Shimadzu, Japan). The organic acid standard solutions were prepared into a series of concentration with the mobile phase. And according to the regression equations of each organic acids that acquired from the analysis of the HPLC figured up the content of organic acids of the rice wine.

The analytical column used throughout the analyses was a Kromasil (Eka Chemicals Company, Sweden) RP-18, 5 µm, 250 mm × 4.6 mm i.d. Cartridge. The solvent A of the mobile phase was made up of 0.01 M monopotassium phosphate (prepared daily with HPLC ultra-pure water, precisely adjusted to pH 2.9 with phosphoric acid). Solvent B was pure acetonitrile. The flow rate was 0.08 mL/min and the ratio of mobile phase A to mobile phase B was 90 %: 10 % (v:v). The eluate was monitored at 210 nm with UV detector.

### 2.5 The determination of volatile substances

The volatile substances of Chinese rice wine were extracted by headspace solid phase micro-extraction (HS-HSPE). The ultrasonic-treated rice wine stored for different days and untreated wine (5 mL each) were placed in 25 mL vials with 2 g NaCl respectively and 20 µL (69 mg/L) styrene. The vials was sealed and preheated at 50℃for 15 min. A CAR-PDMS extraction fibre (Supelco, USA) was inserted into the vials and fractionated from the sample matrix at 50 °C in a thermal block for 40 min until the equilibration of volatiles. Then, the fibre was removed and inserted immediately into an injection port of a gas chromatograph (GC 6890, Agilent, USA) and desorbed for 5 min at 250 °C.

The qualitative analyses of volatile compounds were carried out on a gas chromatography mass spectrophotometer (GC 6890/MS 5973, Agilent, USA) using a DB-WAX column (60 m × 0.25 mm × 0.25 µm, Supelco, USA). The carrier gas was Helium with a flow rate of 0.8 mL/min. Oven temperature program was started from 40 °C and remained for 2 min, to 80 °C with an increase of 3 °C/min and stayed for 3 min, then increasing by 6 °C/min until oven temperature reached 220 °C and holding for 5 min. The injection temperature was 250 °C.

The parameters of the mass spectrophotometer were: interface temperature, 230 °C; ion source temperature, 230 °C; electron impact (EI) spectra obtained at 70 eV; filament current, 200 µA; electrode stem source temperature, 350 °C; scanning mass range of 33–350 *m*/*z*. The identification of volatile compounds was achieved by compared with the mass spectra library of National Institute of Standards of Technology (NIST05, Hewlett-Packard, MD, USA). And qualitative analyses of volatile substances assumed that the efficiency of the volatile substances in the rice wine was the same as that of the internal standard, then the relative content of each volatile substance was calculated from the semi-quantitative method by comparing the percentage of peak areas.

### 2.6 Determination of hydroxyl radical in ultrasound treated sample by fluorescence spectrophotometer

The previous studies [26], [27], [28], [29] show that the terephthalic acid itself is a non-fluorescent substance, but it can react with a hydroxyl radical formed during water sonolysis, and forms 2-hydroxyterephthalate ions which are stable and readily detected using fluorescence spectroscopy with an excitation and emission wavelengths.

A certain volume of terephthalic acid (1 × 10^-4^ mol/L) solution was prepared to sonicate at the condition of ultrasonic density 50 W/L and 40 kHz for 15 min at 30 °C. Periodically during sonication (0, 5, 10, 15, 20 min), samples were removed and the absorbance intensity of each sample was measured using a spectrophotometer (Cary Eclipse, VARIAN Company America). The absorbance values were measured in the individual samples immediately after sonication at an excitation wavelength of 315 nm.

### 2.7 Determination of ultrasonic cavitation yield by UV–Visible spectrophotometer

Ultrasonic cavitation yields are expressed by the amount of acoustic chemical reactions produced by sonicating to the solution. In this paper, the iodine release method was used to measure the acoustic yield in the ultrasonic treatment [30], [31], [32]. Iodine release method refers to the preparation of potassium iodide solution, which contains a certain dissolved air, coupling with a certain amount of starch [33]. Then iodine ions can be transformed into iodine molecules stoichemically under ultrasonic treatment, and the iodine element encountered starch turns blue. Thus the acoustic cavitation yield can be measured by measuring the change in UV absorbance.

Added 10 g/L starch in the 0.2 mol/L potassium iodide solution, and then sonicated at the condition of ultrasonic density 50 W/L and 40 kHz for 15 min at 30 °C. During the sonication, a certain amount of the solution was taken out at 0, 5, 10, 15 and 20 min to determine the change in the absorbance of the solution (wavelength 354 nm) using an UV–Visible spectrophotometry (Beijing Purkinje General Instrument Co., Ltd., Beijing, China). Determination of iodine release was done immediately after sonication for every sample. The intensity of the ultrasonic cavitation effect was reflected by measuring the amount of iodine formed by the potassium iodide.

### 2.8 Detection of free radicals using an electron paramagnetic resonance (EPR) spectrometer

Conditions of EPR measurement are as following:

Test environment temperature: room temperature; Central magnetic field: 3520.000 G; Resolution: 1024 points; Scanning width: 100,000 G; Microwave power: 1.106 mW; Microwave frequency: 9.852 GHz; Adjust frequency:100.00 kHz; Amplitude modulation: 1.00 G; Conversion time: 28.000 ms; Time constant: 10.240 ms; Scanning time: 28.672 s.

Study on the effect of ultrasound time on free radical content: 1) Using quartz capillary to extract pure DMPO solution, measure electron paramagnetic resonance spectrum; 2) Take 20 µL of wine sample and 60 µL of pure DMPO solution and add them to a 1.5 mL centrifuge tube, sample with a quartz capillary tube, and measure the electron paramagnetic resonance spectrum; 3) Use rice wine separately ultrasonic treated for 5, 10, 15, and 20 min, take 20 µL of wine sample and 60 µL of pure DMPO. Add the solution to a 1.5 mL centrifuge tube, take a sample using a quartz capillary, and measure the electron paramagnetic resonance spectrum.

### 2.9 Statistical analysis

Data are presented as mean ± SD (three replicates). Difference between means was compared using Tukey's test and *P* values < 0.05 were regarded as significant. Statistical analysis was conducted using Minitab 2018 software (Minitab Inc., State College, PA, USA).

## 3 Results and discussion

### 3.1 Effects of ultrasonic on the contents of total acids and total esters in Chinese rice wine

As shown in Table 1, the contents of total acids and total esters of rice wine increased by 2.05 % and 7.33 % when sonicated by fixed 40 kHz frequency ultrasonic wave, which had the largest increase and indicated a significant different compared with the untreated rice wine (*P<*0.05). The results were in agreement with research of Zheng et al. [34]. They found that the concentration of total acids and total esters increased in the ultrasonic treated steeped greengage wine. The increase of total acids could be explained by the oxidation of alcohols and aldehydes and the formation of acids in wines because of the dissociation of oxygen that caused by cavitation bubble collapse under ultrasonic conditions [35]. The enhanced esterification effect between alcohols and acids under ultrasonic treatment has also been observed by Ince et al. [36], demonstrating that acids were reacted with alcohols to the formation of ester, which resulted to an increase in total esters content.

**Table 1:** Effects of ultrasonic mode on the content of total acids and esters in Chinese rice wine.

Frequency (kHz)	Total acids (g/L)	Total esters (g/L)
*Untreated*	13.61 ± 0.035^a^	3.41 ± 0.055^a^
20 ± 2	13.73 ± 0.013^a^	3.62 ± 0.063^b^
28 ± 2	13.62 ± 0.035^b^	3.48 ± 0.222^ab^
40 ± 2	13.63 ± 0.030^a^	3.65 ± 0.116^b^
20	13.68 ± 0.030^a^	3.53 ± 0.124^b^
28	13.71 ± 0.042^b^	3.56 ± 0.057^b^
40	13.89 ± 0.031b^c^	3.66 ± 0.075^c^
20/28/40	13.69 ± 0.075^ab^	3.54 ± 0.063^b^
20/28	13.73 ± 0.081^b^	3.61 ± 0.122^bc^
20/40	13.80 ± 0.021^c^	3.65 ± 0.047^c^
28/40	13.72 ± 0.057^b^	3.55 ± 0.129^b^

Different letters in the same line indicate significant difference (*P<*0.05).

Mean values ± standard deviations (n = 3).

According to the results of the total acids and total esters contents between the ultrasonic-treated Chinese rice wine and the untreated, conclusions could be drawn that the fixed single frequency of 40 kHz indicated the significant influence on the maturation of rice wine. Therefore taken the single-frequency ultrasound of 40 kHz as the best ultrasonic frequency to measure the total acids, total esters, organ acids and volatile substances of Chinese rice wine stored for different days, to investigate the effect of ultrasonic cavitation to wine maturation. In this research, results showed that single frequency treatment was better, the possible reason may be that in single frequency treatment, the intensity on unit area of a ultrasonic chamber is higher than two or three frequencies, since the total power are same for one, two or three frequencies. Therefore, it may be speculated that the intensity on unit area transducer is an important factor in ultrasonic treatment.

### 3.2 Effects of ultrasonic on the content of total acids in Chinese rice wine stored for different days

**Fig. 2:**
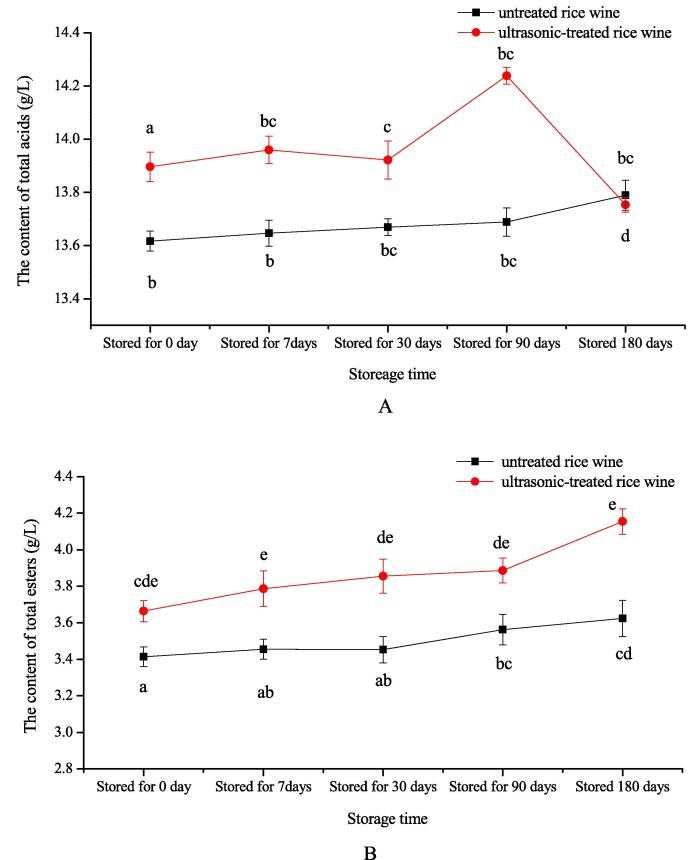
Effects of ultrasonic on the content of total acids (A) and total esters (B) in Chinese rice wine stored for different days. Each value represents mean ± standard deviation of three replicates. The same index marked with different letter means significant difference (*P* < 0.05, n = 3).

For the untreated rice wine, with the increase of the storage time, the content of total acids increased all the time, but there was no significant difference, while for the ultrasonic-treated rice wine, the total acids increased within the first trimester but then decreased to the content that was similar to the untreated rice wine (Fig. 2A). The content of total acids increased the maximum by 4.01 % when sonicated stored for 3 months. Although acids was reacted with alcohols to the formation of ester, alcohols were first oxidized as aldehydes, and eventually oxidized into acids, the content of total acids of rice wine increased gradually as time passed, and in the subsequent storage process, when the total acids content reaches a certain level, it tends to the direction of esterification reaction, leading to a decrease in acids content. [37].

### 3.3 Effects of ultrasonic on the content of total esters in Chinese rice wine stored for different days

As can be seen from the Fig. 2B, the total esters content tended to increase as the time went on, but there was no significant difference whether or not it was subjected to sonication. With time passed by, the content of total esters increased by 9.60 %, 11.66 %, 9.08 %, 14.65 % respectively than the untreated rice wine when remained a week, a month, three months and six months. As time progressed, acids was reacted with alcohols to the formation of ester, which resulted to an increase in total esters content. Similar results were also observed by Shu [38] who stated that total esters, increased with the storage time increased. The lactic acid was the main organic acid of rice wine, accounted for 60 % to 70 % of the total acids from the test data analysis, resulting that the main ester was ethyl lactate, followed by ethyl acetate, ethyl caproate. Ultrasound-assisted wine aging mainly occurs through the ultrasonic rupture of tiny bubbles in the liquid caused by the cavitation effect, which causes the local instantaneous temperature and pressure rise, generating free radicals and triggering a series of complex chemical reactions among the wine compounds, potentially accelerating chemical reactions related to wine aging [15].

### 3.4 Effects of ultrasonic on the content of organ acids in Chinese rice wine stored for different days

In this study, HPLC successfully quantified 8 organ acids in six wine samples (Table 2).

**Table 2:** Changes of organic acids content of rice wine in storage after fixed 40 kHz ultrasonication (mg/L).

Contents (g/L)	Untreated rice wine (Stored for 180 days)	Ultrasonic-treated rice wine				
		Stored for 0 day	Stored for 7 days	Stored for 30 days	Stored for 90 days	Stored for 180 days
Oxalic acid	125.88 ± 6.84^c^	120.50 ± 4.69^ab^	119.27 ± 8.08^a^	124.30 ± 5.99^c^	123.75 ± 4.89^bc^	127.77 ± 5.96^c^
Tartaric acid	127.05 ± 9.09^ab^	123.51 ± 1.25^a^	125.65 ± 2.33^ab^	124.73 ± 3.14^a^	126.05 ± 3.63^ab^	128.95 ± 1.80^b^
Ketoglutaric acid	50.81 ± 5.11^a^	51.37 ± 4.00^a^	51.74 ± 2.94^a^	51.05 ± 3.58^a^	50.35 ± 3.68^a^	50.91 ± 7.96^a^
Citric acid	321.77 ± 23.00^a^	325.30 ± 16.57^a^	327.68 ± 6.32^a^	323.27 ± 43.31^a^	318.80 ± 29.44^a^	322.38 ± 34.47^a^
Malic acid	234.09 ± 10.61^a^	229.41 ± 15.54^a^	234.36 ± 10.18^a^	230.14 ± 9.73^a^	233.00 ± 2.70^a^	235.70 ± 3.54^a^
Succinic acid	187.62 ± 11.71^a^	189.32 ± 4.66^a^	188.16 ± 7.64^a^	197.33 ± 7.91^a^	198.45 ± 13.78^b^	189.86 ± 9.77^a^
Lactic acid	1799.90± 15.18^a^	1829.05± 28.80^a^	1898.75± 12.92^ab^	1873.91± 19.07^ab^	2007.76± 19.86^ab^	2070.60± 18.97^b^
Acetic acid	2337.34± 46.03^b^	2180.98± 58.08^a^	2193.36± 32.39^a^	2184.17± 40.79^a^	2218.11 ± 34.86^a^	2265.39± 32.99^ab^

Different letters in the same line indicate significant difference (*P<*0.05).

Mean values ± standard deviations (n = 3).

As can be seen from the Table 2, in general, the content of measured organic acids was acetic acid > lactic acid > citric acid > malic acid > succinic acid > tartaric acid > oxalic acid > ketoglutaric acid. Among the 8 kinds of organic acids, the contents of lactic acid and acetic acid were relatively high, when compared to the untreated rice wine, acetic acid was decreased by 3.08 % and lactic acid increased by 15.05 % after sonication and stord for six months, which showed an obvious change. However, the other six organic acids did not change significantly, whether compared with the untreated wine or the ultrasonic-treated wine stored for different days.

The total amount of organic acids of untreated rice wine was 5184.47 mg/L, while of the ultrasonic-treated rice wine was 5049.44 mg/L, 5138.99 mg/L, 5108.91 mg/L, 5276.27 mg/L and 5391.56 mg/L when stored for 0 day, 7 days, 30 days, 90 days and 180 days. With the increase of the time after sonication, there was a gradual increase trend of the total amount of organic acids as well as the content of acetic acid and lactic acid, whereas there was no significant difference in the contents of ketoglutaric acid, citric acid and malic acid. Researchers [12], [25], [38] found that the total concentration of organic acids in the best ultrasonic rice wine was not significantly different from that of the new wine, but the total concentration of organic acids in fresh wine and the best ultrasonic rice wine were significantly different compared with 1 year old rice wine. This might be due to the fact that acids were formed by a large number of alcohols and aldehydes through the oxidation reaction during the aging process.

### 3.5 Effects of ultrasonic on the content of volatile substances in Chinese rice wine stored for different days

The contents of volatile components in ultrasonic treated wine and untreated wines determined by GC–MS were shown in Table 3. About 34 kinds of alcohols, 8 kinds of acids, 24 kinds of esters, and 17 kinds of aldehydes were determined in the rice wine. The relative concentrations of volatile substances of the six wine samples were 793.23 mg/L, 1008.99 mg/L, 778.49 mg/L, 873.71 mg/L, 925.74 mg/L and 1112.36 mg/L, respectively. In general, alcohols and esters contents were relatively more than the contents of acids and aldehydes, which was consistent with the effect of ultrasound on the volatile matter of rice wine studied by Hao [12] and Shu [38].

**Table 3:** GC–MS analyse of the contents and varieties of volatile compounds of rice wine in storage after fixed 40 kHz ultrasonication (mg/L).

Violate substances	Untreated rice wine (Stored for 180 days)	Ultrasonic-treated rice wine				
		Stored for 0 day	Stored for 7 days	Stored for 30 days	Stored for 90 days	Stored for 180 days
**Alcohols**	479.12	536.6	353.01	404.2	396.78	417.06
Ethanol	52.85 ± 3.12	46.06 ± 4.67	45.75 ± 2.45	42.85 ± 4.76	37.85 ± 2.39	32.95 ± 2.39
Propanol	62.86 ± 2.32	45.61 ± 2.34	44.83 ± 4.56	42.76 ± 3.58	38.79 ± 3.71	35.45 ± 1.45
Isopropyl alcohol	ND	ND	ND	ND	ND	64.27 ± 3.86
Isobutanol	63.35 ± 3.15	45.97 ± 3.45	44.25 ± 1.76	41.21 ± 3.49	38.75 ± 2.43	35.78 ± 3.12
N-butanol	ND	47.07 ± 2.89	ND	8.68 ± 0.89	ND	ND
N-hexanol	ND	ND	ND	ND	ND	64.91 ± 3.78
Isoamyl alcohol	54.64 ± 4.21	40.62 ± 3.76	15.66 ± 1.34	7.81 ± 0.67	3.45 ± 0.78	ND
1-pentanol	ND	ND	ND	63.26 ± 3.87	ND	ND
2-pentanol	ND	46.65 ± 3.56	ND	64.29 ± 4.21	71.05 ± 4.30	62.76 ± 5.21
2-heptanol	64.31 ± 2.56	ND	ND	ND	ND	ND
Diisobutylmethanol	ND	48.11 ± 4.29	ND	ND	ND	ND
Diethylene glycol	ND	ND	6.86 ± 0.78	ND	ND	ND
Triethylene glycol	ND	46.71 ± 3.85	ND	ND	64.37 ± 5.48	64.33 ± 4.67
Benzyl alcohol	58.06 ± 4.87	55.72 ± 3.45	51.66 ± 3.29	42.36 ± 3.45	31.69 ± 3.24	21.66 ± 2.49
Phenylethanol	6.74 ± 0.98	ND	ND	ND	ND	ND
3-ethoxypropanol	ND	0.46 ± 0.067	ND	ND	ND	ND
3-methylthiopropanol	ND	8.60 ± 0.25	ND	ND	ND	ND
1,3-dimethoxy-2-propanol	6.87 ± 0.56 ND	ND	ND	ND	ND	6.89 ± 0.54
1-amino-2-propanol	0.41 ± 0.034	ND	ND	ND	ND	ND
3-methoxy-1,2-propanediol	6.65 ± 0.76	4.82 ± 0.76	7.65 ± 0.56	64.30 ± 4.32	70.93 ± 5.12	84.50 ± 5.36
1,2-propanediol	ND	ND	6.69 ± 0.87	6.69 ± 0.12	ND	ND
2-methyl-1-butanol	ND	ND	65.08 ± 4.23	ND	ND	ND
4-methoxy-1-butanol	44.33 ± 2.34	ND	ND	ND	ND	ND
4-methyl-1-pentanol	ND	6.31 ± 0.32	ND	ND	ND	ND
4-methyl-4-heptanol	ND	ND	ND	ND	ND	67.84 ± 4.54
3-ethyl-2-methyl-3-pentanol	ND	ND	ND	ND	ND	6.90 ± 0.32
1,3-butanediol	54.27 ± 3.59	46.64 ± 3.24	44.27 ± 3.35	36.12 ± 2.18	ND	ND
2,3-butanediol	ND	46.62 ± 2.34	64.27 ± 4.21	69.29 ± 3.29	74.25 ± 3.78	64.56 ± 3.25
4,4-dimethyl-2-pentanol	ND	ND	ND	ND	ND	9.70 ± -0.18
S-(−)-2-methyl-1-butanol	ND	0.63 ± 0.013	0.87 ± 0.012	ND	ND	ND
3,6,9,12-tetraoxahexadecan-1-ol	3.78 ± 0.19	ND	ND	ND	ND	ND
2-tetradecanol	ND	ND	ND	54.29 ± 4.39	ND	ND
Tetraethylene glycol	ND	ND	ND	6.91 ± 0.27	7.89 ± 0.23	6.95 ± 0.76
**Acids**	39.56	103.33	107.77	66.33	71.48	46.33
Acetic acid	32.84 ± 0.18	46.07 ± 1.23	63.50 ± 4.25	66.33 ± 3.67	52.84 ± 4.36	46.33 ± 2.63
3-aminoisobutyric acid	6.72 ± 0.26	4.87 ± 0.48	0.40 ± 0.023	ND	ND	ND
2-(trimethylsilyl) acetic acid	ND	12.19 ± 0.45	16.80 ± 0.89	ND	ND	ND
Benzoic acid	ND	40.20 ± 2.34	ND	ND	ND	ND
3-nitropropionic acid	ND	ND	6.63 ± 0.67	ND	ND	ND
Neodecanoic acid	ND	ND	20.44 ± 1.76	ND	ND	ND
Thioglycolic acid	ND	ND	ND	ND	11.79 ± 0.78	ND
Ketoglutaric acid	ND	ND	ND	ND	6.85 ± 0.58	ND
**Esters**	171.06	229	253.33	334.81	393.11	661.63
Ethyl formate	ND	ND	ND	ND	0.60 ± 0.026	0.71 ± 0.043
Ethyl acetate	33.68 ± 3.23	46.21 ± 2.78	63.32 ± 4.32	73.83 ± 4.98	79.37 ± 5.32	85.68 ± 5.23
N-hexanoate	43.89 ± 3.89	15.00 ± 0.39	67.89 ± 5.29	67.89 ± 3.78	67.88 ± 4.54	71.89 ± 4.38
Butyrate	ND	ND	ND	14.10 ± 0.12	62.69 ± 4.32	66.41 ± 4.47
Ethyl isobutyrate	ND	ND	ND	ND	63.74 ± 3.45	68.79 ± 4.67
Ethyl pentanoate	ND	45.51 ± 3.48	ND	ND	52.72 ± 4.26	62.72 ± 3.78
Isovalerate	ND	ND	ND	ND	ND	33.82 ± 3.46
Ethyl heptanoate	ND	ND	ND	ND	ND	47.88 ± 4.36
Octanoate	ND	ND	ND	62.72 ± 4.32	67.88 ± 2.39	67.88 ± 4.78
Ethyl lactate	54.25 ± 4.34	64.81 ± 3.45	66.63 ± 4.34	73.56 ± 4.38	76.43 ± 428	84.25 ± 2.35
Isoamyl acetate	ND	2.61 ± 0.19	ND	ND	13.72 ± 1.25	35.92 ± 3.54
Ethyl dodecanoate	ND	ND	17.92 ± 1.02	ND	ND	ND
Triethyl borate	ND	ND	14.35 ± 0.67	15.25 ± 0.78	26.37 ± 1.38	24.24 ± 3.23
Succinic acid monoethyl ester	ND	18.54 ± 1.45	ND	ND	25.56 ± 2.32	28.56 ± 3.38
Diethyl succinate	18.88 ± 1.43	20.44 ± 1.23	25.32 ± 2.32	26.86 ± 1.89	30.82 ± 1.67	34.42 ± 2.19
Ethyl glycolate	ND	ND	ND	0.60 ± 0.023	ND	ND
Phenethyl acetate	8.95 ± 0.21	ND	12.21 ± 1.09	18.92 ± 1.45	11.95 ± 0.98	ND
Ethyl 3-phenylpropionate	8.99 ± 0.39	ND	ND	ND	ND	ND
Diisobutyl phthalate	1.62 ± 0.87	ND	ND	ND	ND	ND
Dibutyl phthalate	ND	ND	ND	ND	ND	1.36 ± 0.098
Ethyl palmitate	0.80 ± 0.062	ND	ND	ND	ND	ND
γ-butyrolactone	ND	15.88 ± 1.34	23.22 ± 1.87	ND	ND	29.29 ± 1.28
Ethyl phenylacetate	ND	ND	ND	ND	ND	17.96 ± 1.38
**Aldehydes**	103.49	140.06	64.39	68.37	64.37	33.67
Acetaldehyde	32.94 ± 2.34	25.49 ± 2.19	12.94 ± 2.28	10.94 ± 0.73	0.25 ± 0.012	ND
Butyraldehyde	ND	ND	ND	ND	2.20 ± 0.039	ND
Hexanaldehyde	ND	ND	14.14 ± 0.91	ND	6.26 ± 0.27	3.14 ± 0.21
Nonanal	ND	47.21 ± 2.45	ND	ND	ND	ND
Isobutyraldehyde	ND	ND	ND	ND	ND	1.58 ± 0.042
Isovaleraldehyde	ND	ND	ND	ND	4.11 ± 0.18	2.15 ± 0.038
Dimethyl formal	4.35 ± 0.21	0.70 ± 0.034	ND	ND	ND	ND
Glycerol formaldehyde	6.84 ± 0.21	ND	ND	ND	ND	ND
Furfural	28.62 ± 1.29	19.79 ± 1.42	ND	18.12 ± 0.54	14.26 ± 0.19	8.52 ± 0.35
Benzaldehyde	27.14 ± 1.38	18.72 ± 1.54	18.24 ± 1.08	39.31 ± 2.12	29.23 ± 1.27	15.21 ± 0.28
Phenylacetaldehyde	ND	9.39 ± 0.19	ND	ND	8.06 ± 0.29	ND
8-hydroxyquinoline-2-carbaldehyde	3.60 ± 0.31	ND	ND	ND	ND	ND
2-methyl butyraldehyde	ND	ND	ND	ND	ND	3.07 ± 0.045
Isobutyraldehyde diacetal	ND	18.76 ± 1.04	ND	ND	ND	ND
Acetaldehyde ethylhexyl acetal	ND	ND	14.79 ± 0.71	ND	ND	ND
Triacetaldehyde	ND	ND	4.28 ± 0.074	ND	ND	ND
**Total**	793.23	1008.99	778.49	873.71	925.74	1112.36

ND means “No Found”.

Mean values ± standard deviations (n = 3).

Referring to conventional parameters, a general decreasing trend was observed in alcohol content in terms of ageing status. The relative contents of volatile alcohols of the six wine samples were 479.12 mg/L, 536.6 mg/L, 353.01 mg/L, 404.2 mg/L, 396.78 mg/L and 417.06 mg/L. With the time increased, the contents of ethanol, propanol, isobutanol, isoamyl alcohol and phenylethanol in ultrasonic-treated rice wine decreased gradually.

The relative concentrations of volatile acids in the six kinds of rice wine were 39.56 mg/L, 103.33 mg/L, 107.77 mg/L, 66.33 mg/L, 71.48 mg/L and 46.33 mg/L, respectively. Among them, the highest content was acetic acid. On the whole, the content of volatile acids of ultrasonic-treated rice wine firstly rose and then declined as time went on, while the 3-amino isobutyric acid content was gradually reduced. And only acetic acid was detected in the ultrasonic-treated wine stored for six months. Part of alcohol was oxidized into acetic acids that resulted in an increase in the content of total acids [12], [15], [25].

Esters are one of the main flavour contributors in alcoholic beverages. The relative concentrations of volatile esters in the six kinds of rice wines were 171.06 mg/L, 229 mg/L, 253.33 mg/L, 334.81 mg/L, 393.11 mg/L and 661.63 mg/L, respectively. With the increase of the standing time, the volatile ester content increased gradually. The concentration of ethyl acetate and ethyl lactate were the main compounds of volatiles of rice wine, and the two kinds of volatile esters improved after ultrasonic treatment. In addition, after the sonication, ethyl pentanoate, isoamyl acetate and monoethyl succinate were detected, ethyl isovalerate and ethyl heptanoate were detected only in the wine stored for six months when sonicated. Zheng et al. [34] found that the content of esters in steeped greengage wine increased by 7.74 % after ultrasonic irradiation, suggesting the accelerating effect of esterification.

The relative concentrations of volatile aldehydes in the six kinds of rice wine were 103.49 mg/L, 140.06 mg/L, 64.39 mg/L, 68.37 mg/L, 64.37 mg/L and 33.67 mg/L, respectively. After ultrasonic treatment, most of the aldehydes in rice wine decreased, which reduced the unpleasant offensive flavor. Chang et al. [19] found that, with 20 kHz ultrasonic wave treatments, the levels of acetaldehyde of wine decreased as the level in 1-year conventionally matured maize wine, which made the wine taste better.

In general, when compared to the untreated rice wine that stored for six month, the contents of volatile alcohols, aldehydes, acids and esters in ultrasonic-treated wine were decreased by 12.95 %, 67.46 % and increased by 17.11 %, 286.78 %, respectively. With the increase of storage time, in combination with the prominent increase of the esters and prominent decrease of the alcohols and acetaldehyde, the Chinese rice wine tasted unspicy, mellow, and smooth. Ultrasonic treatment increased the ester content of rice wine and accelerated the maturation process of rice wine.

### 3.6 Determination of hydroxyl radicals in different ultrasonic time with fluorescence spectrometry

**Fig. 3:**
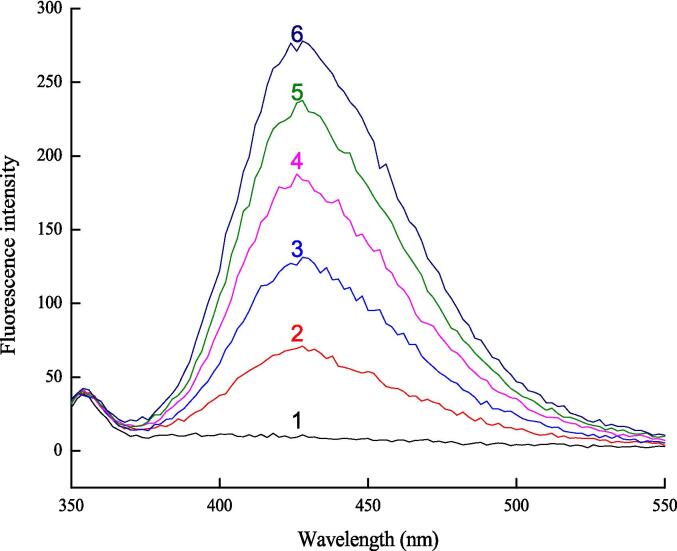
Fluorescence emission spectra of terephthalic acid solution at different ultrasound time (Excitation wavelength: 315 nm). 1- Double Distilled water; 2- Rice wine without sonication; 3- Rice wine sonicated for 5 min; 4- Rice wine sonicated for 10 min; 5- Rice wine sonicated for 15 min; 6- Rice wine sonicated for 20 min.

The fluorescence spectra of terephthalic acid solution were measured before and after sonication (Fig. 3). The results showed that there was no emission fluorescence before sonication, and after sonication, the fluorescence emission spectrum appeared, and gradually increased with the increase of ultrasonic time. Studies [15] showed that the cavitation effect of ultrasound could cause the hydrolysis of water molecules to produce hydroxyl radicals, and the generation of hydroxyl radicals was positively related to sonication time and intensity. That is, with increasing time of sonication or intensity, the more hydroxyl radicals will be generated. Ultrasound can promote association and enhance the affinity between polar molecules, such as ethanol and water, and can even form larger, firmer polar molecules. Ultrasound can reduce the activation energy of certain reactions, then accelerate esterification, condensation, redox and degradation reactions within wine, and improve its alcohol-ester aroma and flavor [15].

### 3.7 Effects of ultrasonic cavitation on the maturation of Chinese rice wine

**Fig. 4:**
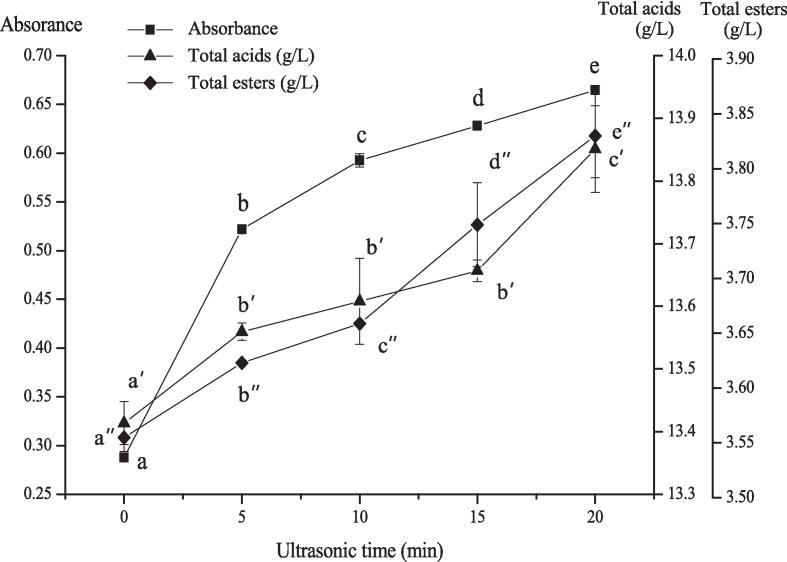
The effect of ultrasonic cavitation on the maturation of the Chinese rice wine. Values are means ± SD (three replicates). Different letters were used to show significant differences among ultrasonic cavitation yields (*P<*0.05).

The intensity of ultrasonic cavitation effect could be reflected by the ultrasonic cavitation yield. Fig. 4 showed the effect of ultrasonic time on cavitation yield, and could also indirectly indicate the effect of ultrasonic cavitation yield on total acids and total esters in rice wine. As shown in Fig. 4 that the release of iodine gradually increased with the increase of ultrasonic time, and the differences were significant among different times (*P<*0.05), meanwhile the total acids and total esters in rice wine also increased with the increase of ultrasonic time, which were consistent with the trend of ultrasonic cavitation yield. It could be explained that to some extent ultrasound was beneficial to improve the quality of rice wine. A series of studies have been carried out and it was found out the existence of pulsed cavitation peaks in the reverberation field. Generally, the chemical effects of ultrasound are due to the phenomenon of acoustic cavitation, which is the growth, oscillations and subsequently violent collapse of numerous tiny bubble nuclei formed from acoustical wave-induced compression/rarefaction in a body of liquid. Studies also found that the production of hydroxyl radicals increased with the increase of ultrasonic output power per unit volume, and the linear relationship between the hydroxyl radicals produced by the ultrasonic wave and the ultrasonic time was the quite well at the optimal output power [15], [35], [39].

### 3.8 EPR test results

**Fig. 5:**
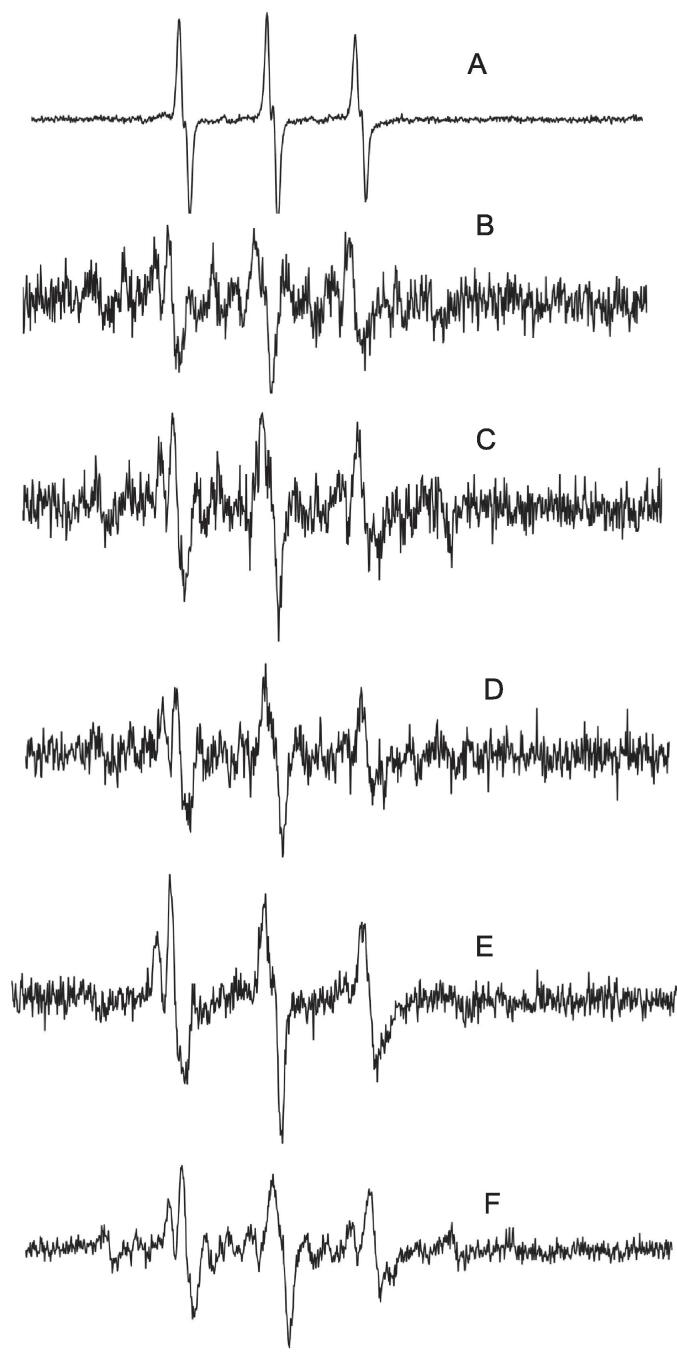
Electron spin resonance spectroscopy analysis of ultrasonic treated samples (A) pure DMPO electron spin resonance spectroscopy, (B) rice wine untreated by ultrasound + pure DMPO electron spin resonance spectroscopy, (C) rice wine after 5 min of ultrasonic treatment + pure DMPO electron spin resonance spectroscopy, (D) rice wine after 10 min of ultrasonic treatment + pure DMPO electron spin resonance spectroscopy, (E) rice wine after 15 min of ultrasonic treatment + pure DMPO electron spin resonance spectroscopy, (F) rice wine after 20 min of ultrasonic treatment + pure DMPO electron spin resonance spectroscopy.

As shown in Fig. 5, DMPO free radical scavenger was used to capture the free radicals generated during the ultrasound process. From Fig. 5C to F, heights of peaks increase as ultrasonic intensity increases, and in Fig. 5G, heights of peaks relatively decline compared to those in Fig. 5F. Based on the analysis of electron spin resonance spectra, it can be concluded that rice wine was undergone natural aging and ultrasound treatment. With ultrasonic treatment, 1-hydroxyethyl radicals will be generated. Kreitman [40] et al. found natural oxidation in red wine model wines. The free radical that plays a major role in the process is also 1-hydroxyethyl free radical. The reason is that ultrasonic cavitation firstly generates hydroxyl radicals, which then react with ethanol in the wine to produce 1-hydroxyethyl radical [25]. It was found that 1-hydroxyethyl free radical was produced in wine under ultrasound treatment, and this radical is a key radical in the maturation process of wine [40], [41], which is the basis for the occurrence of related reactions during wine maturation. The mechanisms of its production may be due to the transient high temperatures and pressures generated locally by ultrasonic cavitation that causes the cleavage of water molecules in wine to produce hydroxyl radicals, which subsequently react with ethanol to form 1-hydroxyethyl radicals [42].

## 4 Conclusion

Till now, most of the current researches on ultrasonic treatment of wine are conducted under laboratory-scale conditions. In the present work, a 20 L scale ultrasonic reactor was used to sonicate Chinese rice wine. And it was found out that the single fixed-frequency 40 kHz ultrasonic showed the significant increases in total acids and total esters (*P<*0.05), which indicated that ultrasonic treatment had a positive effect on the maturation process of Chinese rice wine. In general, with the prolonging of the storage time, the total amount of organic acids and volatile substances displayed gradual increase trends after 40 kHz sonication. The content of volatile esters increased, while the volatile alcohols and aldehydes decreased. It was found that sonication treatment caused a maximum increase of 286.78 % volatile esters compared to control after stored for six months. The mechanism of ultrasound on the quality parameters and sensory characteristics of rice wine have not be studied thoroughly and systematically, theoretical basis for the commercial application of ultrasound aging are needed, especially on the aging acceleration after sonication. The mechanism study of this research demonstrated that, with the ultrasonic time increased, the free radicals and acoustic cavitation yields increased, and the total acids and total esters content correspondingly showed the same growing trend, suggesting that acoustic cavitation might be effective to accelerate the maturation process. This research results on the improvement of properties and maturity of rice wine by scale-up ultrasonic treatment showed good prospects for the technique’s industrial application.

## CRediT authorship contribution statement

**Zhuofan He:** Writing – original draft, Methodology, Investigation, Data curation. **Furong Hou:** Investigation, Data curation. **Yansheng Du:** Data curation. **Chunhua Dai:** Data curation. **Ronghai He:** Writing – review & editing, Supervision, Methodology, Funding acquisition, Conceptualization. **Haile Ma:** Data curation.

## Declaration of competing interest

The authors declare that they have no known competing financial interests or personal relationships that could have appeared to influence the work reported in this paper.
